# Full-length transcriptome analysis of *Phytolacca americana* and its congener *P. icosandra* and gene expression normalization in three Phytolaccaceae species

**DOI:** 10.1186/s12870-020-02608-9

**Published:** 2020-08-27

**Authors:** Danfeng Liu, Li Chen, Chao Chen, Xingkui An, Yongjun Zhang, Yi Wang, Qingjun Li

**Affiliations:** 1 Yunnan Key Laboratory of Plant Reproductive Adaption and Evolutionary Ecology, Yunnan University, Kunming, 650504 China; 2 Laboratory of Ecology and Evolutionary Biology, State Key Laboratory for Conservation and Utilization of Bio-Resources in Yunnan, Yunnan University, Kunming, 650504 China; 3 State Key Laboratory for Biology of Plant Diseases and Insect Pests, Institute of Plant Protection, Chinese Academy of Agricultural Sciences, Beijing, 100191 China

**Keywords:** Phytolaccaceae, SMRT sequencing, Full-length transcriptome analysis, Reference gene evaluation, RT-qPCR

## Abstract

**Background:**

Phytolaccaceae species in China are not only ornamental plants but also perennial herbs that are closely related to human health. However, both large-scale full-length cDNA sequencing and reference gene validation of Phytolaccaceae members are still lacking. Therefore, single-molecule real-time sequencing technology was employed to generate full-length transcriptome in invasive *Phytolacca americana* and non-invasive exotic *P. icosandra*. Based on the transcriptome data, RT-qPCR was employed to evaluate the gene expression stability in the two plant species and another indigenous congener *P. acinosa*.

**Results:**

Total of 19.96 Gb and 19.75 Gb clean reads of *P. americana* and *P. icosandra* were generated, including 200,857 and 208,865 full length non-chimeric (FLNC) reads, respectively. Transcript clustering analysis of FLNC reads identified 89,082 and 98,448 consensus isoforms, including 86,989 and 96,764 high-quality ones. After removing redundant reads, 46,369 and 50,220 transcripts were obtained. Based on structure analysis, total 1675 and 1908 alternative splicing variants, 25,641 and 31,800 simple sequence repeats (SSR) as well as 34,971 and 36,841 complete coding sequences were detected separately. Furthermore, 3574 and 3833 lncRNA were predicted and 41,676 and 45,050 transcripts were annotated respectively. Subsequently, seven reference genes in the two plant species and a native species *P. acinosa* were selected and evaluated by RT-qPCR for gene expression analysis. When tested in different tissues (leaves, stems, roots and flowers), *18S rRNA* showed the highest stability in *P. americana*, whether infested by *Spodoptera litura* or not. *EF2* had the most stable expression in *P. icosandra*, while *EF1-α* was the most appropriate one when attacked by *S. litura*. *EF1-α* showed the highest stability in *P.acinosa*, whereas *GAPDH* was recommended when infested by *S. litura*. Moreover, *EF1-α* was the most stable one among the three plant species whenever germinating seeds or flowers only were considered.

**Conclusion:**

Full-length transcriptome of *P. americana* and *P. icosandra* were produced individually. Based on the transcriptome data, the expression stability of seven candidate reference genes under different experimental conditions was evaluated. These results would facilitate further exploration of functional and comparative genomic studies in Phytolaccaceae and provide insights into invasion success of *P. americana*.

## Background

*Phytolacca americana* is a member of the Phytolaccaceae family and is native to northeast America. Because of its ornamental and medicinal applications, it was introduced into China in 1935 [1]. Unfortunately, it has evolved into an invasive species and spread to most areas of the country, especially in central and southern China. Compared to non-invasive exotic congener *P. icosandra* and native congener *P. acinosa*, *P. americana* is of interest because it exhibits multiple biological activities, such as plant pesticides, antimicrobial property, heavy metal accumulation capacity [2–4].

In order to investigate the mechanisms of various bioactivities of *P. americana* further, transcriptome-wide study is necessary to facilitate. Reports have showed that jasmonic acid-induced and cadmium-treated transcriptome data of *P. americana* have been obtained by Illumina HiSeq 2500 and Illumina HiSeq 2000 platform, respectively [5, 6]. However, these data were both achieved by second generation sequencing (SGS), which could not produce full-length transcripts. Genomic data of *P. americana* was available at the SRA under project PRJNA544344, but it’s raw reads without coding sequences prediction and functional annotation [7].

Third generation sequencing (TGS) is known for its kilobase-sized long reads and is an outstanding strategy for better understanding RNA processing. For example, it can be used to analyze different transcript isoforms regulated by alternative splicing, which greatly increases the repertoire of proteins, lead to genetic and functional diversity and is prevalent in most eukaryotic organisms [8]. The long reads could also provide sequence information on gene-coding regions for functional analysis at the transcriptional level and thus can be applied to refine an assembled genome for better annotation [9]. However, TGS could not quantify gene expression for the moment and have a relatively high error rate than SGS. The combination of TGS and SGS are able to solve this problem and are highly recommended by most researchers [10].

With the transcriptome and genome data available, functional genomics research is being performed which relied heavily on gene expression analysis. Reverse transcription quantitative real time PCR (RT-qPCR) has been reported to be a very sensitive and accurate technique to analyze gene expression level, but it requires appropriate reference gene as an internal control to normalize mRNA levels between different samples for an exact comparison of gene expression [11, 12]. An ideal reference gene should be expressed at a constant level across various experimental conditions. However, studies have shown that no single reference gene is universal for all experimental conditions [13–15]. Therefore, it’s necessary to estimate the stability of reference genes under particular experimental condition before using them for gene expression analysis.

In the present study, to provide high-quality and more complete assemblies in genome and transcriptome studies of Phytolaccaceae, a hybrid sequencing approach combining the SGS and TGS technologies was carried out. First, full-length transcriptome of the invasive plant species of *P.ameracana* and an non-invasive exotic congener *P. icosandra* was generated by single-molecule real-time (SMRT) sequencing. Alternative splicing events, simple sequence repeats (SSR), coding sequences, protein annotations and long non-coding sequences were analyzed respectively at transcription level. Further, the stability of reference genes was evaluated in two Phytolaccaceae species mentioned above and one native congener *P. acinosa* by RT-qPCR in order to facilitate future research on functional gene expression.

## Results

To classify the plant species, these three Phytolaccaceae members, *P. americana, P. icosandra and P.acinosa* were identified by PCR and followed by sequences alignment based on sequences of second internal transcribed spacer (*ITS2*) and the intergenic spacer of photosystem II protein D1 gene and tRNA-His gene of chloroplast genome (*psbA-trnH*) (Table S1). The sequences of *ITS2* and *psbA-trnH* in *P. americana* that we employed had the identity of 100% with the sequences reported by Chen [16]. In *P. icosandra*, the sequences of *ITS2* had 99.5% identity and the sequences of *psbA-trnH* had 95.7% identity with the results of Chen’s [16]. In *P.acinosa*, 100% similarity of *ITS2* and 99.4% identify of *psbA-trnH* were found [16].

### SMRT sequencing data output

Using the Pacific Biosciences’ SMRT sequencing protocol, 19.96 Gb clean reads of invasive species *P. americana* were obtained after preprocessing. On the basis of full passes > = 3 and sequence quality > 0.9, 235,097 circular consensus sequences (CCS) with 85.44% full-length rate were obtained, including 200,857 full-length non-chimeric (FLNC) sequences and 86,989 high-quality consensus isoforms. After removing redundant sequences from the high quality consensus isoforms, 46,369 transcripts, 1675 alternative splicing events, 25,641 SSR, 34,971 complete coding sequences, 3574 lnc RNAs and 41,676 annotated transcripts in *P. americana* were achieved. Similarly, 19.75 Gb clean reads in *P. icosandra* were identified, and 223,203 CCS with 89.56% full-length rate, 208,865 FLNC sequences as well as 96,764 high-quality consensus isoforms were filtered. Subsequently, 50,220 transcripts and 1908 alternative splicing events were obtained. What’s more, 31,800 SSRs, 36,841 complete coding sequences, 3833 lnc RNAs and 45,050 annotated transcripts were identified in *P. icosandra* (Table 1).

**Table 1:** Summary of full-length transcriptome sequencing

	*P. americana*	*P. icosandra*
Clean reads (Gb)	19.96	19.75
CCS	235,097	233,203
FLNC	200,857	208,865
FLNC (%) (FLNC/CCS)	85.44	89.56
Consensus isoform	89,082	98,448
High quality consensus isoform	86,989	96,764
Transcripts	46,369	50,220
Alternative splicing	1675	1908
SSR	25,641	31,800
Complete coding sequences	34,971	36,841
LncRNA	3574	3833
Annotated transcripts	41,676	45,050

### Transcriptome analysis

Based on the structure of achieved transcripts, 1675 and 1908 alternative splicing events were identified in *P. americana* and *P. icosandra*, respectively. Transcripts of 43,942 (100,469,039 bp in total) in *P. americana* were employed for SSR analysis based on the sequence length that was more than 500 bp, including 25,641 SSRs and 17,231 SSR-containing sequences. Similarly, 48,070 transcripts (117,904,499 bp in total) in *P. icosandra* were employed for SSR analysis, and 31,800 SSRs together with 20,155 SSR-containing sequences were identified. The detail information about the number of sequences containing more than one SSR, the number of SSRs present in compound formation, and the number of different types of SSRs were shown in Table 2. In addition, total of 34,971 complete coding sequences (CDS) in *P. americana* and 36,841 CDS in *P. icosandra* were identified by using TransDecoder. The length distribution of predicted proteins was shown in Fig. S1.

**Table 2:** SSRs obtained from transcripts with more than 500 bp

Searching item	*P. americana*	*P. icosandra*
Total number of sequences examined	43,942	48,070
Total size of examined sequences (bp)	100,469,039	117,904,499
Total number of identified SSRs	25,641	31,800
Number of SSR containing sequences	17,231	20,155
Number of sequences containing more than 1 SSR	5641	7229
Number of SSRs present in compound formation	2635	3571
Number of mono nucleotide SSR	12,699	16,366
Number of di nucleotide SSR	7182	8065
Number of tri nucleotide SSR	5266	6593
Number of tetra nucleotide SSR	192	283
Number of penta nucleotide SSR	82	121
Number of hexa nucleotide SSR	220	372

**Fig. 1:**
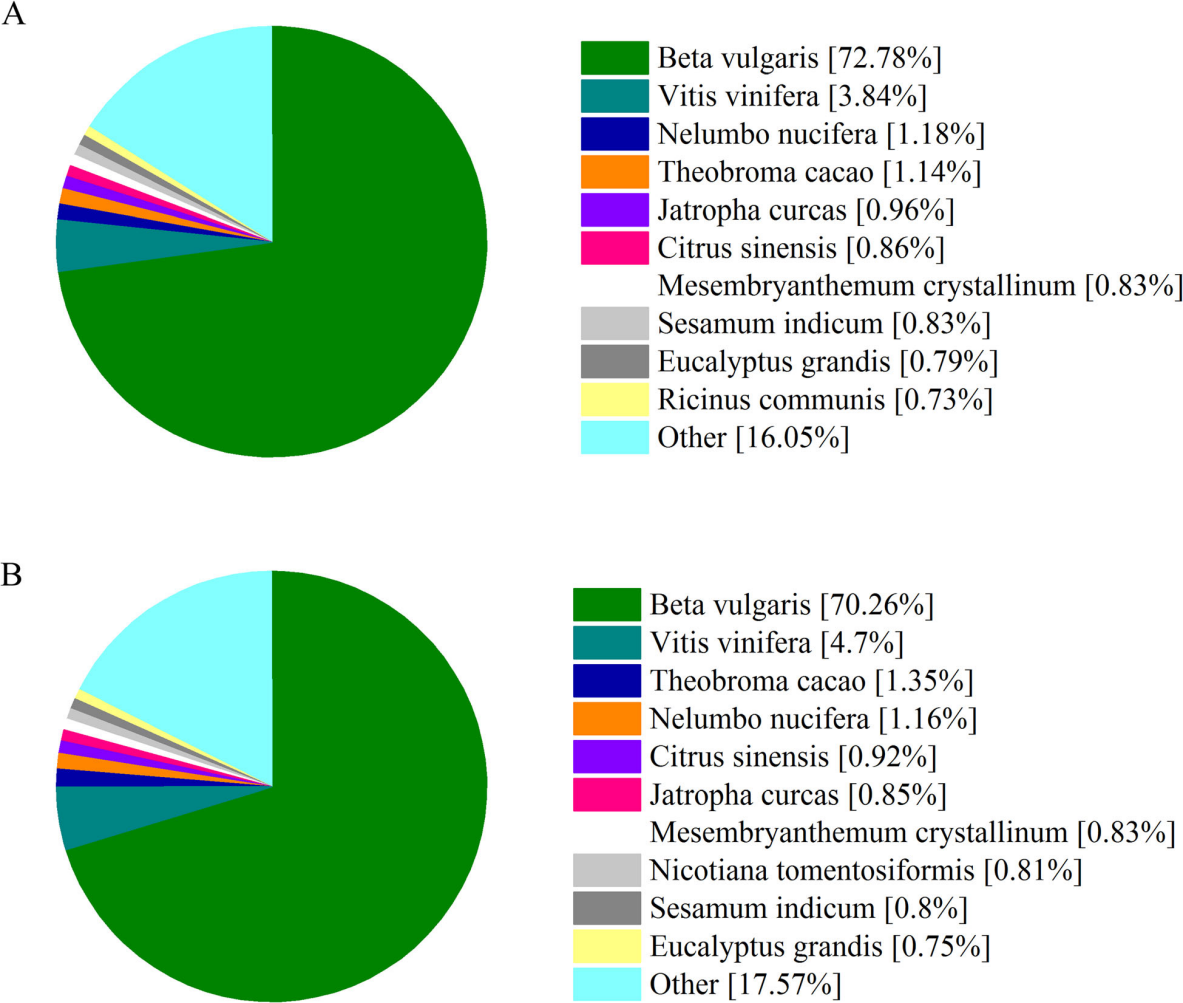
Homologous species distribution of *P. americana* and *P. icosandra* annotated based on the Nr database. **a**, *P. americana*; **b**, *P. icosandra*

**Fig. 2:**
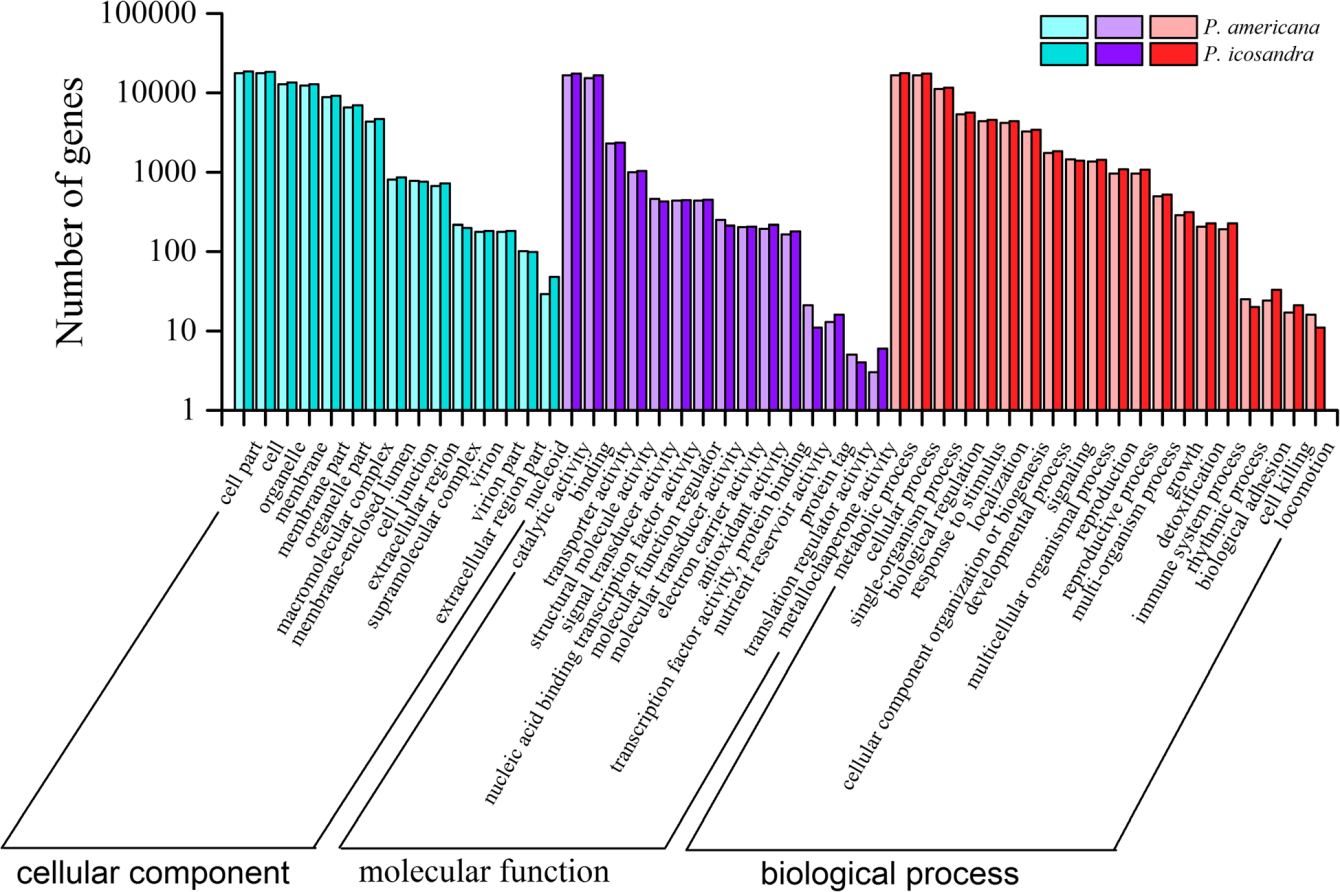
Classification of the transcripts annotated by the Gene Ontology (GO)

With the eight protein databases, sequence alignments were performed to annotate predicted proteins. In total, 41,676 transcripts in *P. americana* and 45,050 transcripts in *P. icosandra* were annotated separately (Table 3). The number of annotated protein sequences in *P. americana* was similar with *P. icosandra* under a particular database. Specifically, Nr (NCBI non-redundant protein) analysis revealed that approximately 30,000 transcripts in *P. americana* and 31,000 transcripts in *P. icosandra* showed the highest sequence similarity with *Beta vulgaris* (Fig. 1). GO (Gene Ontology) assignment also suggested that similar amount of sequences in the two plant species belonged to the same term. And many were classified into cell part and cell term of cellular component, catalytic activity and binding of molecular function, and metabolic process and cellular process of biological process (Fig. 2). COG (Clusters of Orthologous Groups of proteins) annotation showed that a large number of predicted proteins in the two plant species were linked to functional class R (General function prediction only), J (Translation, ribosomal structure and biogenesis), T (Signal transduction mechanisms), G (Carbohydrate transport and metabolism) and O (Posttranslational modification, protein turnover, chaperones) (Fig. S2). The result of eggNOG (evolutionary genealogy of genes: Non-supervised Orthologous Group) annotation indicated that most of the annotated proteins in the two plant species were belonging to the functional class S (Function unknown) (Fig. S3). KOG (euKaryotic Ortholog Groups) functional classification suggested that R (General function prediction only) and O (Posttranslational modification, protein turnover and chaperones) were the most abundant functional categories in the two plant species (Fig. S4). These results indicated that most of the sequences obtained were truly functional proteins and had a similar functional classification in *P. americana* and its congener *P.icosandra*. Even though more work is needed to identify sequences that regulated or involved in the invasion success of *P. americana*, the annotation of predicted proteins provided necessary information for further studies.

**Table 3:** Number of proteins annotated via differential protein database

Databases	*P. americana*			*P. icosandra*		
	Annotated Number	300 ≤ length < 1000	Length ≥ 1000	Annotated Number	300 ≤ length < 1000	Length ≥ 1000
COG	16,701	1494	15,197	17,249	1496	15,742
GO	31,055	4171	26,838	32,733	4157	28,535
KEGG	17,017	2191	14,798	18,130	2211	15,893
KOG	26,396	2853	23,508	28,948	2933	25,979
Pfam	32,822	3571	29,239	34,684	3708	30,963
Swiss-Prot	29,608	3503	26,055	31,485	3524	27,920
eggNOG	40,363	5251	35,043	43,423	5262	38,109
Nr	41,485	5612	35,784	44,692	5590	39,026
All	41,676	5699	35,884	45,050	5705	39,267

**Fig. 3:**
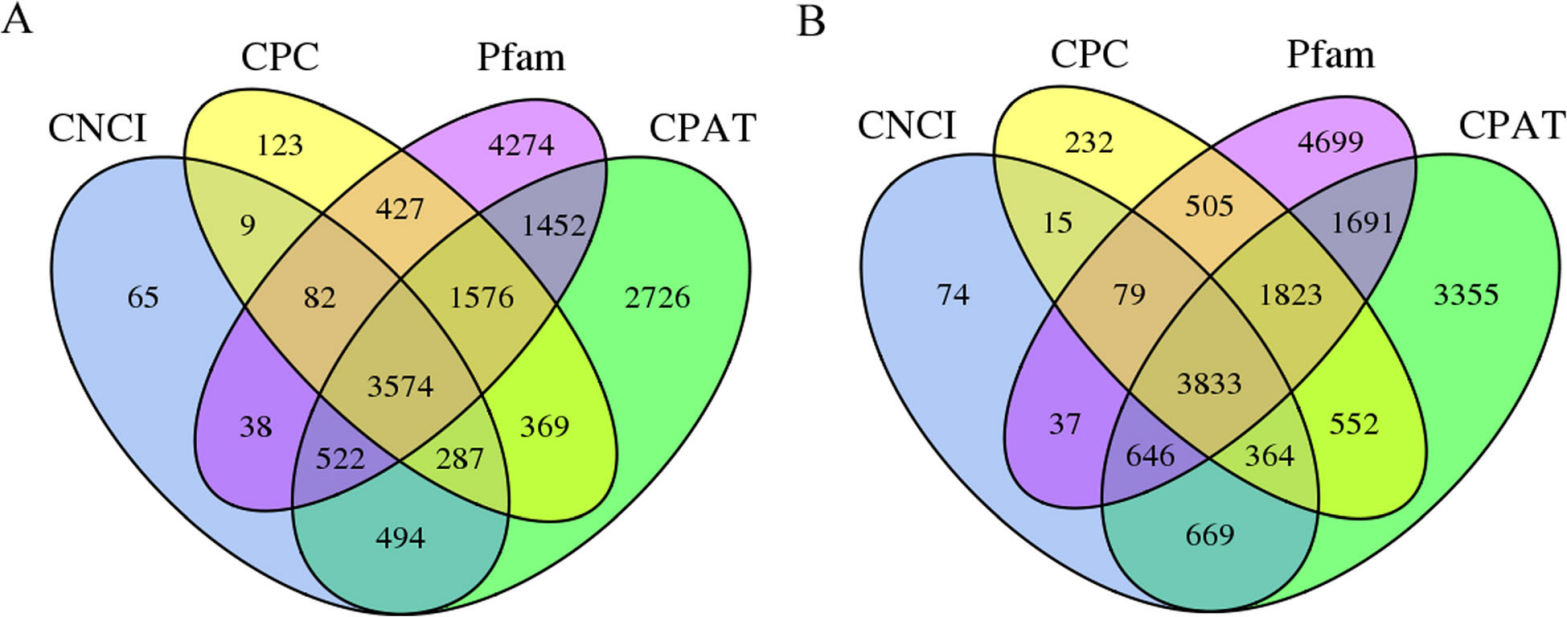
Venn diagram of the number of lncRNAs predicted by CPC, CNCI, CPAT and Pfam. **a**, *P. americana*; **b**, *P. icosandra*

**Fig. 4:**
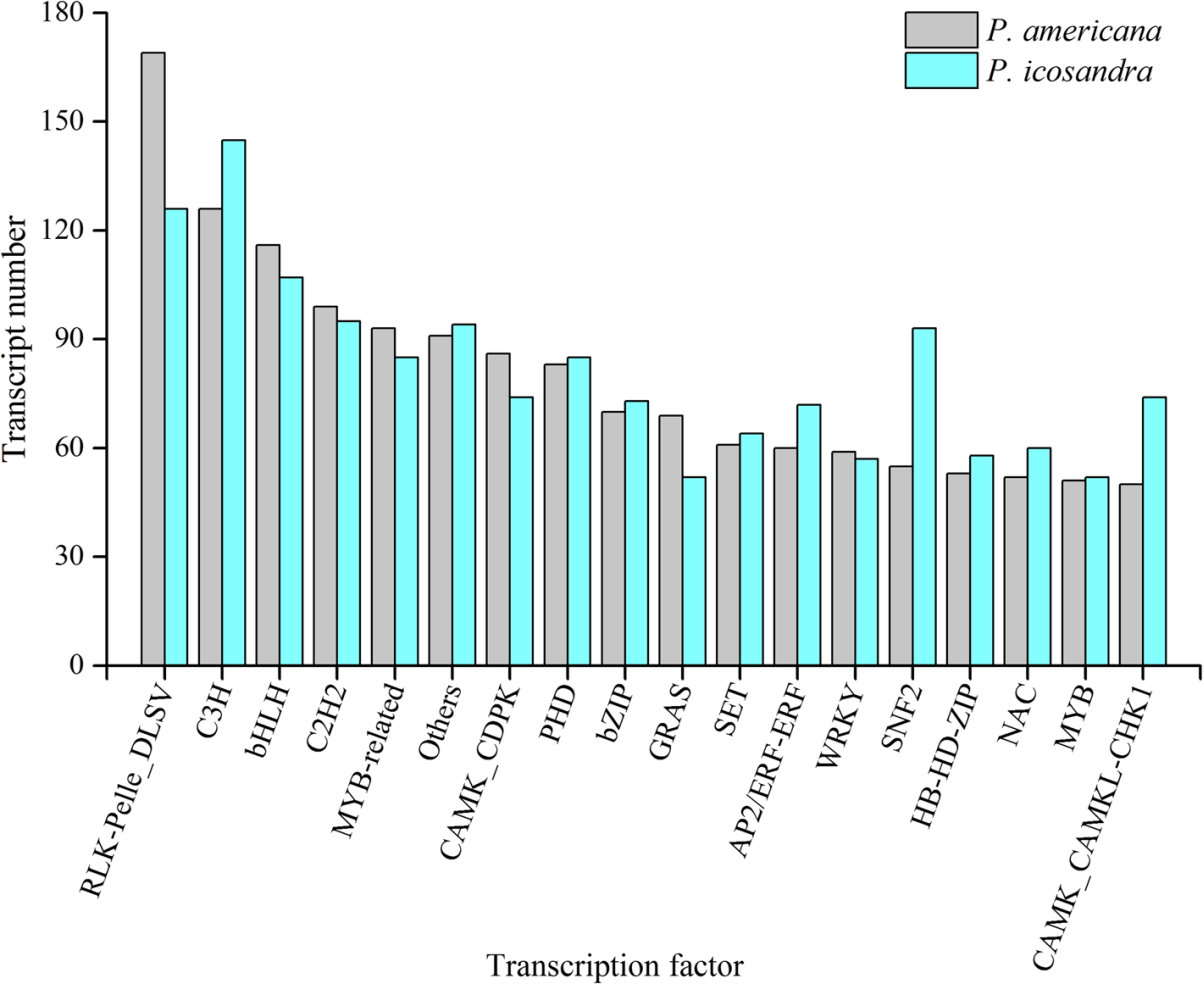
Classification of predicted transcription factors

Besides the transcripts encoding proteins, long non-coding RNAs (lncRNAs) were achieved. LncRNAs are reported to be key regulators in plant biological processes. The number of lncRNA in *P.ameracana* and *P.icosandra* was predicted by CPC (Coding Potential Calculator), CNCI (Coding-Non-Coding Index), Pfam and CPAT (Coding Potential Assessment Tool), respectively. In total, 3574 lncRNA in *P.americana* and 3833 lncRNA in *P.icosandra* were predicted by all these four methods (Fig. 3). Subsequently, transcription factors (TFs) that are key components involved in the transcriptional regulatory system were predicted. In *P. americana*, 3991 TFs of 204 types were filtered. And in *P. icosandra*, 4022 TFs of 201 types were predicted. These two plant species shared the first 10 types of TFs, but the number of each type TF was not similar, especially RLK-Pelle_DLSV, C3H, SNF2 and CAMK_CAMKL-CHK1, indicating the particular functions on transcript regulation (Fig. 4).

### Amplification performance of RT-qPCR

Primers designed for RT-qPCR were evaluated by PCR first. The primers which produced single amplicon without primer dimer were chosen for melting curve analysis. Only primers which produced a single fragment were chosen for qPCR amplification efficiency assessment. The qPCR efficiency of each primer pair was generated from a 10-fold serial dilution of pooled cDNA and was shown in Table 4.

**Table 4:** Primers for RT-qPCR analysis

Gene name	Gene description	Primer sequence (5′-3′)	Length (bp)	PCR Efficiency (%)	R^2^
*Tubulin*	Tubulin 8	F: GTAAGGAAGCCGAGAATTG	181	103	0.984
		R: TCAACAACAGTGTCAGAGA			
*EF1-α*	Elongation factor 1-alpha	F: TGAAGAAGGTCGGATACAAT	194	95	0.993
		R: GTAGACATCCTGGAGTGG			
*GAPHD*	Glyceraldehyde-3-phosphate dehydrogenase	F: TGGTGCTAAGAAGGTTATTATC	198	104	0.986
		R: GAGTGAACGGTGGTCATA			
*EF2*	Elongation factor 2	F: GTATCACCATCAAGTCAACTG	194	99	0.999
		R: ACAATCAACCACAACAAGG			
*18S rRNA*	18S rRNA	F: ACTTCCTCTTCTCGTATCATT	193	99	0.993
		R: TGTTCAGCATAGACTGTGA			
*β-Actin*	Actin-7	F: ATGCTATCCTTCGTCTGG	180	105	0.996
		R: TACTCTTGGCTGTCTCTG			
*28S rRNA*	28S rRNA	F: TACGATTGGTTACGGACAT	191	102	0.983
		R: TTCTCATCAACAACAGCATAT			

R^2^, linear regression coefficient

**Fig. 5:**
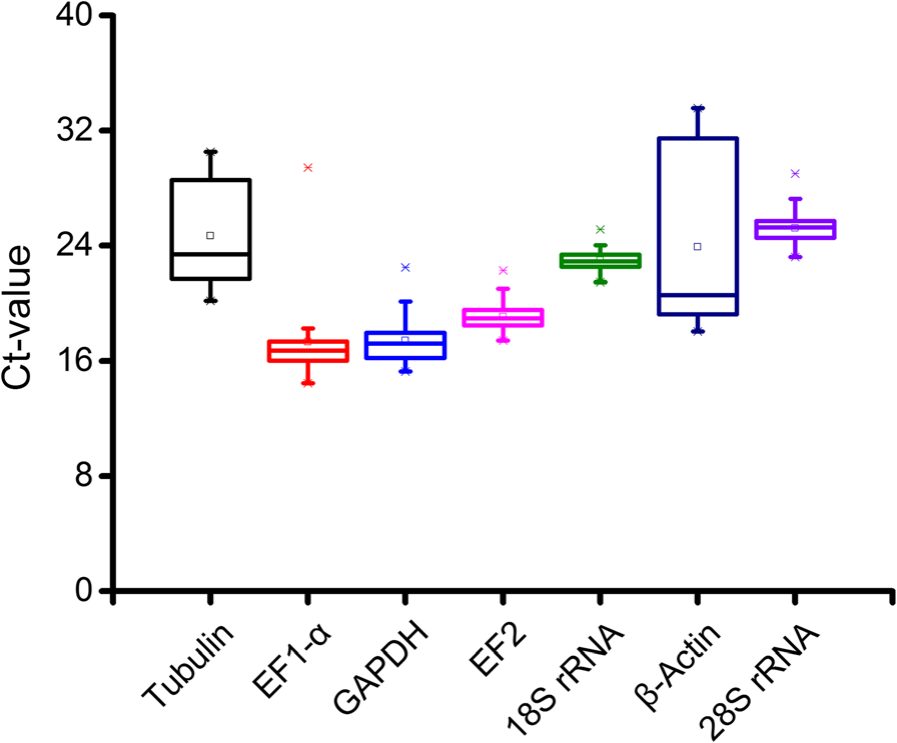
RNA transcription levels of seven candidate reference genes in *P. americana*, *P.icosandra* and *P. acinosa*. The expression level of candidate reference genes in total samples (*n* = 99) was presented as cycle threshold number (Ct-value) and explained by box and whisker plots. The asterisks represented the minimum and maximum Ct value. The squares indicated the 25th and 75th percentiles, and the median was represented by a bar across the square

The threshold cycle (Ct) values of each reference gene were employed to evaluate expression level under different experimental conditions (Fig. 5). Average Ct values for all the seven candidate reference genes ranged from 17.3 to 25.2, in which *EF1-α* showed the highest expression level and *28S rRNA* had the lowest expression level. It was also suggested that Ct values of *β-Actin* and *tubulin* fluctuated significantly across all the experimental samples.

### Stability of candidate reference genes

To determine the appropriate reference genes for normalization in different experimental conditions, the expression data was analyzed by geNorm, NormFinder and BestKeeper, respectively (Table S2).

**Fig. 6:**
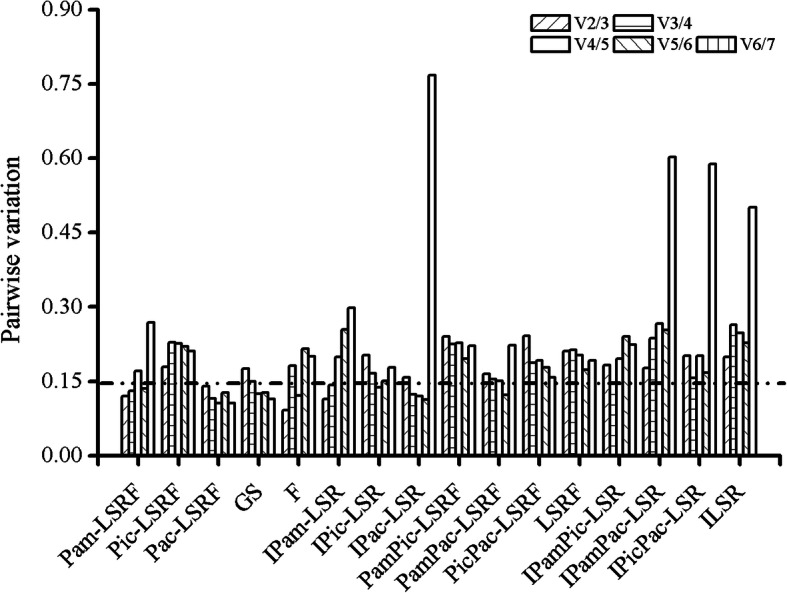
Pairwise variation analyzed by geNorm to determine the optimal number of reference genes for accurate normalization. A threshold value of 0.15 was suggested for valid normalization. If the value of V_n/n + 1_ (pairwise variation) is less than 0.15, then n reference genes in combination are recommended for gene normalization. If the value of V_n/n + 1_ is more than 0.15, then V_n + 1/n + 2_ should be taken into account. Pam: *P. americana*; Pic: *P. icosandra*; Pac: *P. acinosa*; LSRF: different tissues of leaves, stems, roots and flowers; GS: germinating seeds of these three plant species; F: flowers of these three plant species; LSR: different tissues of leaves, stems and roots; I: infested by *S. litura* of third instar

When expression stability of reference genes were analyzed in different tissues (leaves, stems, roots and flowers) of *P. americana*, *18S rRNA* and *EF2* of *P.americana* were identified as the most suitable reference genes by geNorm and NormFinder, and *18S rRNA* was also suggested by BestKeeper. Pairwise variation value of V_2/3_ was below the cutoff value of 0.15, which means the combination of two reference genes were most suitable for gene expression normalization (Fig. 6). When tested in *P.icosandra*, *EF1-α* was recommended for normalizing gene expression analysis not only by geNorm but also by NormFinder. *EF2* was also suggested by geNorm and BestKeeper. In *P.acinosa*, *EF1-α* was the best reference gene suggested by geNorm and BestKeeper, but *18S rRNA* was recommended by NormFinder. The use of two reference genes was suitable because pairwise variation value of V_2/3_ was below 0.15. When pooled the data of different tissues from *P.americana* and *P.icosandra* together, *EF2* was shown to be the most stable gene by all the three methods. When investigated the expression stability of reference genes in different tissues of *P.americana* and *P.acinosa*, *18S rRNA* showed the best expression stability by geNorm and NormFinder, while *EF2* was referred as the most stable one by BestKeeper. However, the combination of five reference genes was recommended by geNorm for V_5/6_ which was less than 0.15. When the data of different tissues from *P.icosandra* and *P.acinosa* was put together, *EF1-α* was identified as the best one by geNorm and NormFinder, whereas *EF2* was suggested to be the best stability reference gene by BestKeeper. When set the data of these three plant species as a pool, *EF1-α* was suggested to be the most stable one by geNorm and NormFinder, while *EF2* was also recommended by geNorm and BestKeeper. According to these results, it is very important to select the appropriate reference gene when analyze the gene expression level among plant species.

When analyzed the data among germinating seeds, *28S rRNA* and *EF1-α* were identified as the best reference genes by geNorm, while *18S rRNA* was recommended by NormFinder, and *GAPDH* was suggested by BestKeeper. Three reference genes were sufficient to normalize gene expression for V_3/4_ was below 0.15. In flowers only of these three plant species, *EF1-α* was confirmed by all the three methods. The geNorm analysis showed that the value of V_4/5_ was below 0.15. So, four reference genes in combination were suggested. These results indicated that when focusing on particular tissues of different plant species, the selection of reference gene was also very essential.

When plants were infested by *S. litura*, *18S rRNA* showed the most expression stability suggested by geNorm and NormFinder in different tissues of *P.americana*, while *EF1-α* was revealed by BestKeeper. The combination of two reference genes was suggested by geNorm due to the value of V_2/3_ was less than 0.15. *18S rRNA* was also recommended by geNorm in *S. litura*-infested *P.icosandra*, and *EF1-α* was shown to be the most stable one by NormFinder and BestKeeper. Four reference genes in combination were recommended by geNorm. *18S rRNA* was also identified as the best reference gene by geNorm in plant species *P.acinosa*, while *Tubulin* was suggested by NormFinder and *28S rRNA* was recommended by BestKeeper. The combination of three reference genes was appropriate by geNorm. When analyzed the data of two plant species together, *18S rRNA* showed the best stability in geNorm and NormFinder, while the expression stability of *EF1-α* was suggested by BestKeeper in *P.americana* and *P.icosandra*. *18S rRNA* was identified as the best reference gene by all the three algorithms in *P.americana* and *P.acinosa*. *18S rRNA* and *β-Actin* were suggested by geNorm in *P.icosandra* and *P.acinosa*, while *GAPDH* and *EF2* were recommended by NormFinder and BestKeeper, respectively. When take all the data of *S. litura*-infested plant species into account, *18S rRNA* exhibited the most stable expression suggested by geNorm and NormFinder, while *EF2* was the gene with the most constant expression identified by BestKeeper.

## Discussion

Full-length transcripts are fundamental resources for structural, functional and comparative genomics research [9, 17]. SMRT sequencing has been acknowledged by enabling the generation of multikilobase sequences to improve genome and transcriptome assembly [18]. The full-length cDNA sequences generated are able to characterize the post-transcriptional process, such as alternative splicing, lncRNA prediction, and coding sequences for further gene functional studies. Based on the full-length transcriptome data generated, about 20 Gb of clean data were obtained for *P.americana* and *P.icosandra*, respectively (Table 1). Accordingly, the number of CCS, FLNC, consensus isoforms, high-quality isoforms, transcripts, alternative splicing events, SSRs, complete coding sequeces, lncRNAs and annotated transcripts were analyzed, providing basic transcriptomic information for further studies.

Reports have showed that full-length transcriptome of *Zea mays* have greatly helped in refining gene annotation and revealed the complexity of gene expression in maize [9]. Similar analysis has also been conducted in *Sorghum bicolor* [17]. What’s more, the world expansion capability of *Cydia pomonella* has been informed according to its genome information [19]. Molecular mechanism of rapid growth and invasive adaptation of an invasive species *Mikania micrantha* has also been investigated according to its reference genome [20]. Therefore, the full-length transcriptome data of *P.americana* and *P.icosandra* will contribute to the genomic research and provide insights into invasive mechanism of *P.americana* through comparative genomics study in Phytolaccaceae species.

Accurate relative quantification of RT-qPCR for further gene expression analysis relies on robust normalization by stably expressed reference genes to minimize error in the experimental process [21]. Therefore, suitable reference genes for the normalization of relative gene expression data in three Phytolaccaceae species, *P.americana*, *P.icosandra* and *P.acinosa*, were sought under a diverse set of conditions. These results demonstrated the importance of validating reference genes under the relevant experimental conditions. For example, in different tissues (leaves, stems, roots and flowers) of *P.americana*, *18S rRNA* and *EF2* were recommended to be the best-suited reference genes, and similar results were found in *S. litura*-infested *P.americana*. However, even though the appropriate reference genes in *P.icosandra* were ranked according to the analyzed results of the three methods, all the pairwise variation values were above the cutoff value of 0.15, while the combination of *18S rRNA*, *β-Actin*, *EF1α* and *EF2* were most suitable in *S. litura*-infested *P.icosandra*. *EF2* and *EF1α* have been considered as the ideal reference genes in *P.acinosa*, whereas the combination of *18S rRNA*, *β-Actin* and *GAPDH* were recommended after *S. litura* infestation. Researches have also showed that no single reference gene is stably expressed among different tissues of an organism, such as the reference gene selection in *Amygdalus persica*, *Solanum lycopersicum* and *Glycine max* [11, 13, 14]. What’s more, our results also suggested that reference genes identified based on transcriptome data should be confirmed by experimental evidence. In JA-induced transcriptome of *P. americana*, *28S rRNA* showed stable expression between exogenous JA-treated and control plants [5]. JA signal pathway of plants can be induced by Lepidopteran herbivores infestation [22]. However, *18S rRNA* and *EF2* were identified as the most stable expression reference genes in *P. americana* after *S. litura* infestation.

In order to conduct the gene expression analysis among different plant species of Phytolaccaceae, the data of the three plant species were also compared together. When compared the data in germinating seeds of three plant species, various genes were recommended by the three methods. The combination of plant species under other experimental conditions showed that the pairwise values of almost all the combination were higher than the cutoff value of 0.15, except the combination of *P.americana* and *P.acinosa* where five reference genes were recommended for data normalization as well as the combination of *S. litura*-infested *P.americana* and *S. litura*-infested *P.icosandra* where three reference genes were suggested. These results indicated that no particular gene was expressed constantly across different plant species, even though these plants are congeners. Therefore, reference genes should be employed appropriately under the relevant experimental conditions.

## Conclusion

The research has provided transcriptome-wide full-length isoforms of *P.americana* and *P.icosandra*, providing insights into invasive success of *P.americana.* Guidelines for selecting appropriate reference genes under different tissues in one plant species or among varied plant species were recommended further. No particular gene was expressed constantly under different experimental conditions indicating the necessity of reference gene identification. These results would facilitate the exploration of functional and comparative genomics studies in Phytolaccaceae to better understand plant biology.

## Methods

### Plant and insect materials

Plants of *P. americana* (24°49′N, 102°52′E), *P. icosandra* (25°08′N, 102°44′E) and *P. acinosa* (25°26′N, 104°19′E) used in this study, which was named M, K and Q first, were collected in Yunnan, China. Sampling was permitted when conducted complying with local legislation. The formal identification of the samples were conducted by Chao Chen, botany major of Laboratory of Ecology and Evolutionary Biology, State Key Laboratory for Conservation and Utilization of Bio-Resources in Yunnan, Yunnan University, according to *Flora of China* (Vol.5:435–436, 2013), *Flora of North America* (Vol.4:3–11, 2003), Chinese Virtual Herbarium (http://www.cvh.ac.cn/) and Global Plants on JSTOR (http://plants.jstor.org/). DNA identification was also employed according to the *ITS2* region of nuclear ribosomal DNA, one of the most widely used DNA fragments in plant molecular systematics at the generic and species levels, and the chloroplast *psbA-trnH* intergenic region [16]. All voucher specimens were maintained at an experimental field of Laboratory of Ecology and Evolutionary Biology, State Key Laboratory for Conservation and Utilization of Bio-Resources in Yunnan, Yunnan University.

Tissues of leaves, stems, roots and flowers from one individual plant of *P. americana* or *P. icosandra* were collected individually from the wild in Yunnan province, and no permission is needed for collecting theses samples. Each sample was flash frozen in liquid nitrogen and stored at − 80 °C for further experiments.

Third instar larvae of *Spodoptera litura* were purchased from Henan Jiyuan Baiyun Industry Co., Ltd., China (shop101732681.taobao.com) and then were reared on artificial diet in a climate chamber (14 h at 27 °C with light and 10 h at 25 °C without light) for further use.

For reference gene evaluation, seeds of *P. americana*, *P. icosandra* and *P. acinosa* were collected first from the wild in Yunnan province, and no permission is needed. The seeds were sown separately in 1% agar plates and cultivated in the climate chamber. After 5 d, five germinating seeds of one plant species were collected together as one sample for subsequent experiments. Each plant species have three replications. Two weeks later, other germinating seeds of each species were transplanted into plastic pots (15 cm diameter and 12.5 cm height) with soil (Jiangsu Peilei Matrix Technology Development Co., Ltd., China), and cultivated with adequate water in artificial chambers with same conditions as described above. Four months later, leaves, stems, roots and flowers of each plant species were collected individually. Simultaneously, six larvae *S. litura* of third instar were employed to infest on *P. americana*, *P. icosandra* or *P. acinosa* with one insect per leaf. Control treatments were herbivore free. After 24 h infestation, leaves, stems and roots of these three plant species were harvested individually. All samples collected were flash frozen in liquid nitrogen and stored in − 80 °C for subsequent assays, and three replicates were conducted for each treatment.

### Nucleic acid extraction and assays

Genomic DNA was isolated from the leaves of different plant species following protocols provided by DNAquick Plant System (Tiangen Biotech Co., Ltd., Beijing, China). Then it was employed as the PCR template for plant species identification.

Total RNAs from different tissues was prepared using RNAprep Pure Plant Kit (Polysaccharides & Polyphenolics-rich; Tiangen Biotech Co., Ltd., Beijing, China) according to the manufacturer’s instructions. The RNA quality and purity were measured by using a NanoPhotometer N60 (Implen, Germany) and the Agilent Bioanalyzer 2100 system (Agilent Technologies, CA, USA). Samples only with a 280/260 ratio of 1.9 to 2.1, a 230/260 ratio between 2.0 and 2.4 and a RIN value more than 7.0 were chosen for the sequencing library construction. An equal amount of total RNAs from four different tissues of the same plant species were mixed as one sample for full-length transcriptome sequencing.

Total RNAs from the samples collected for reference gene evaluation was also extracted individually as described above. For each sample, cDNA was prepared by using 1.5 μg of total RNA following the recommended instructions of FastQuant RT kit (with gDNase; Tiangen Biotech Co., Ltd., Beijing, China).

### PacBio cDNA library preparation and SMRT sequencing

Full-length cDNA was synthesized by using the SMARTer™ PCR cDNA Synthesis Kit (Clontech, CA, USA). The generated cDNA was then re-amplified using PCR. After end repairing, SMRT adaptor with a hairpin loop structure was ligated to the cDNA. Via exonuclease digesting, the cDNA library was constructed. After quality measurement of the cDNA library, SMRT sequencing was performed using the Pacific Bioscience Sequel platform following the provided protocol.

### Illumina cDNA library construction and second-genaration sequencing

The extracted mRNA was purified using oligo (dT)-attached magnetic beads. Fragmentation was conducted in the NEBNext First Strand Synthesis Reaction Buffer. First-strand cDNA was acquired based on the random hexamers, and then the second-strand cDNA was synthesized with dNTPs, RNase H and PrimeSTAR GXL DNA polymerase. The synthesized cDNA was purified with AMPure XP beads. After end repairing, adding poly A and adaptor ligation, AMPure XP beads were used for size selection. The generated cDNA was then amplified for building cDNA libraries. The qualified libraries were pair end sequenced on Illumina nova platform.

### Quality filtering and error correction of long reads

Raw SMRT sequencing reads were filtered by removing polymerase reads less than 50 bp and sequence accuracy less than 0.9. After removing adaptor, subreads were obtained. Clean data was produced with subreads more than 50 bp. CCSs were produced from clean data with parameters of full passes > = 3 and accuracy over 0.9. After examining the co-existence of 5′ and 3′ adaptors and poly (A) tail, full-length transcripts were selected. During the processes of library preparation, the chimeric sequences formed by the direct linkage of two cDNA template strands due to the low concentrations of adaptor or SMRTbell are called artificial chimeric sequences. The non-chimeric sequences in the full-length transcripts are the full-length non-chimeric (FLNC) sequences.

As SMRT sequencing generates a high error rate, it is necessary to perform error correction. Iterative clustering was used first to obtain consensus isoforms, and the full-length consensus sequences from iterative clustering for error correction were refined using Quiver [9, 10]. Moreover, the raw Illumina SGS reads were filtered to remove adaptor sequences and low quality reads, and error correction of low-quality isoforms was conducted using the SGS reads with the software Proovread [23]. In briefly, the short reads of Illumina RNA-Seq data were mapped to the low quality isoforms, and then the base in the low quality isoform was replaced by the particular base that had the maximum number of occurrences during the mapping. Full-length consensus isoforms with post-correction accuracy of > 99% (high-quality consensus isoform) were generated for further analysis. Any redundancy in high-quality full-length sequences was removed by CD-HIT [24].

### Transcriptome analysis

#### Alternative splicing detection

RNA alternative splicing occurs in a pre-mRNA transcript, which leads to a single gene encoding multiple proteins and therefore increases transcriptome and proteome diversity and plays a key role in regulating gene expression and protein function [8]. In this study, alternative splicing isoforms were screened as described in SMRT sequencing of *Agasicles hygrophila* [25].

#### SSR detection

SSRs, also known as microsatellites, are tandem repeats (typically 5–50 times) of certain DNA motifs ranging in length from 2 bp to 13 bp [26]. According to the transcriptome data, sequences that were more than 500 bp were chosen for SSR analysis. Seven SSR types, mononucleotide, dinucleotide, trinucleotide, tetranucleotide, pentanucleotide, hexanucleotide and compound SSR, were analyzed using the MIcroSAtellite identification tool (MISA; http://pgrc.ipk-gatersleben.de/misa/http://pgrc.ipk=gatersleben.de/misa/). The result can be employed in plant genetic studies, especially for the study of population genetics and invasion history of invasive plant species *P. americana*.

#### Coding sequences prediction

To predict all the protein-coding sequences, TransDecoder (http://github.com/TransDecoder/TransDecoder/releases) was employed [27]. Subsequently, the number of proteins with different length was calculated.

#### Functional annotation of transcripts

To assign putative gene function, the obtained non-redundant transcripts were aligned to eight protein databases by BLASTx with e-value < 10^− 5^, and the retrieved proteins with the highest sequence similarity with the given transcript was used for protein functional annotation. These databases included Nr, KEGG (Kyoto Encyclopedia of Genes and Genomes), SwissProt, COG, GO, KOG, Pfam and eggNOG [25].

#### Long non-coding RNA (lncRNA) prediction

LncRNA is an interesting topic in biology and have been found to be essential regulators in a wide spectrum of biological processes such as gene expression regulation, control of translation or imprinting [28]. Therefore, CPC, CNCI, CPAT and Pfam protein structure domain analysis were employed to filter coding potential of the transcripts and then screen lncRNAs.

### Candidate reference genes selection, qPCR and data analysis

For reference genes evaluation, seven of the commonly used candidate genes (*Tublin*; *Elongation factor 1-α*, *EF1-α*; *Glyceraldehyde-3-phosphate dehydrogenase*, *GAPDH*; *Elongation factor 2*, *EF2*; *18S rRNA*; *β-Actin*; *28S rRNA*) were chosen based on the annotation of full-length transcriptome data. Primers for the seven reference genes were designed individually by Beacon Designer 7.9 (Premier Biosoft International, CA). The sequences of primers, amplicon length, PCR efficiency and linear regression coefficient were shown in Table 4.

The qPCR measurement was performed on an ABI QuantStudio 7 Flex PCR system (Applied system, Carlsbad, CA). All samples were assayed in 20 μL reaction volume using the SuperReal PreMix Plus kit (SYBR Green; Tiangen Biotech Co., Ltd., Beijing, China) according to the manufacturer’s instruction. The thermal profile of the RT-qPCR reactions was as follows: 15 min at 95 °C, followed by 40 cycles of 10 s at 95 °C and 32 s at 60 °C. Melting curve analysis (60–95 °C) by the end of each PCR reaction was routinely performed to verify primer specificity. Melting curves showed a single amplified product for all genes. All samples were technically tested in triplicate.

Expression levels were determined by the default setting of the Ct value. The relative fold-changes of gene expression between different samples were calculated using the comparative Ct method. Primer amplification efficiencies and standard deviations were calculated on a standard curve generated using a 10-fold dilution series of pooled cDNA over five dilution points. Expression stability of the seven selected reference genes was evaluated by geNorm [29], BestKeeper [30] and NormFinder [31]. And the optimal number of reference genes was also calculated by geNorm [14, 29].

## Supplementary information

**Additional file 1: Table S1.** Identification of three employed Phytolaccaceae species. **Table S2.** Ranking orders of reference genes validated according to three kinds of software. **Figure S1.** Length distribution of protein sequences transcript from the coding sequences predicted. **Figure S2.** Classification of the transcripts annotated by the Clusters of Orthologous Groups of proteins (COG). **Figure S3.** Classification of the transcripts annotated by the evolutionary genealogy of genes: Non-supervised Orthologous Groups (eggNOG). **Figure S4.** Classification of the transcripts annotated by the euKaryotic Ortholog Groups (KOG).

## Data Availability

All data generated during this study are included in this published article and its supplementary information file. All the transcriptome data have been deposited in the NCBI Sequence Read Archive (SRA) under accession number PRJNA613739 and PRJNA613892. The transcriptome data will not be released before Dec. 31, 2021 for further study reason, but is available from the corresponding author on reasonable request.

## Abbreviations

SMRT: Single-molecule real-time
RT-qPCR: Reverse transcription quantitative real time PCR
TGS: Third generation sequencing
SGS: Second generation sequencing
ITS2: The second internal transcribed spacer
CCSs: Circle consensus sequences
FLNC: Full-length non-chimeric
SSR: Simple sequence repeats
CDS: Coding sequences
ORF: Open reading frame
Nr: NCBI non-redundant protein
KEGG: Kyoto Encyclopedia of Genes and Genomes
COG: Clusters of Orthologous Groups of proteins
GO: Gene Ontology
KOG: euKaryotic Ortholog Groups
eggNOG: Evolutionary genealogy of genes: Non-supervised Orthologous Group
LncRNA: Long non-coding RNA
CPC: Coding Potential Calculator
CNCI: Coding-Non-Coding Index
CPAT: Coding Potential Assessment Tool
*EF1-α*: *Elongation factor 1-α*
*GAPDH*: *Glyceraldehyde-3-phosphate dehydrogenase*
*EF2*: *Elongation factor 2*
Ct: Threshold cycle
