# Supplementary material for: Full-length transcriptome analysis of Phytolacca americana and its congener P. icosandra and gene expression normalization in three Phytolaccaceae species
Source: BMC Plant Biol. 2020 Aug 27;20:396. doi: 10.1186/s12870-020-02608-9 (PMC7450566; doi:10.1186/s12870-020-02608-9)
Supplement: Supplementary file 1 — Additional file 1: Table S1. Identification of three employed Phytolaccaceae species. Table S2. Ranking orders of reference genes validated according to three kinds of software. Figure S1. Length distribution of protein sequences transcript from the coding sequences predicted. Figure S2. Classification of the transcripts annotated by the Clusters of Orthologous Groups of proteins (COG). Figure S3. Classification of the transcripts annotated by the evolutionary genealogy of genes: Non-supervised Orthologous Groups (eggNOG). Figure S4. Classification of the transcripts annotated by the euKaryotic Ortholog Groups (KOG). [file 12870_2020_2608_MOESM1_ESM.docx]

**Supplementary material**

**Table S1 Identification of three employed Phytolaccaceae species**

| Plant species | Identity to *ITS2* (%) | Species identified based on *ITS2* | Idetntity to *psbA-trnH* (%) | Species identified based on *psbA-trnH* |
| --- | --- | --- | --- | --- |
| M | 100 | *P. americana* | 100 | *P. americana* |
| K | 99.5 | *P. icosandra* | 95.7 | *P. americana* |
| Q | 100 | *P. acinosa* | 99.4 | *P. acinosa* |

**Table S2 Ranking orders of reference genes validated according to three kinds of software**

| Experimental conditions | rank | geNorm | | NormFinder | | BestKeeper | | | |
| --- | --- | --- | --- | --- | --- | --- | --- | --- | --- |
|  |  | gene name | stability value | gene name | stability value | gene name | r | SD | CV |
| Different tissues of *P.americana* | 1 | 18S rRNA | 0.206 | EF2 | 0.072 | 18S rRNA | 0.96 | 0.52 | 2.29 |
|  | 2 | EF2 | 0.206 | 18S rRNA | 0.072 | β-Actin | 0.94 | 0.79 | 4.05 |
|  | 3 | EF1-α | 0.319 | EF1-α | 0.112 | EF2 | 0.925 | 0.52 | 2.76 |
|  | 4 | β-Actin | 0.438 | β-Actin | 0.151 | EF1-α | 0.901 | 0.43 | 2.57 |
|  | 5 | 28S rRNA | 0.614 | Tubulin | 0.603 | Tubulin | 0.512 | 0.6 | 2.76 |
|  | 6 | Tubulin | 0.715 | 28S rRNA | 0.681 | 28S rRNA | 0.039 | 0.38 | 1.47 |
|  | 7 | GAPDH | 1.059 | GAPDH | 1.296 | GAPDH | 0.974 | 1.98 | 11 |
| Different tissues of *P.icosandra* | 1 | EF1-α | 0.556 | EF1-α | 0.104 | EF2 | 0.943 | 0.97 | 5.16 |
|  | 2 | EF2 | 0.556 | EF2 | 0.147 | 18S rRNA | 0.659 | 0.54 | 2.32 |
|  | 3 | GAPDH | 0.587 | GAPDH | 0.279 | EF1-α | 0.972 | 1.1 | 6.73 |
|  | 4 | 18S rRNA | 0.775 | 18S rRNA | 0.607 | GAPDH | 0.935 | 1.01 | 5.68 |
|  | 5 | β-Actin | 0.95 | β-Actin | 0.763 | β-Actin | 0.93 | 1.49 | 7.17 |
|  | 6 | 28S rRNA | 1.12 | 28S rRNA | 0.967 | 28S rRNA | 0.609 | 1.07 | 4.24 |
|  | 7 | Tubulin | 1.258 | Tubulin | 0.996 | Tubulin | 0.539 | 1.19 | 5.21 |
| Different tissues of *P.acinosa* | 1 | EF1-α | 0.242 | 18S rRNA | 0.039 | EF1-α | 0.961 | 0.61 | 3.67 |
|  | 2 | GAPDH | 0.242 | EF1-α | 0.185 | 18S rRNA | 0.928 | 0.38 | 1.66 |
|  | 3 | Tubulin | 0.372 | Tubulin | 0.307 | EF2 | 0.883 | 0.76 | 3.9 |
|  | 4 | 18S rRNA | 0.44 | GAPDH | 0.351 | GAPDH | 0.851 | 0.71 | 4.22 |
|  | 5 | 28S rRNA | 0.498 | 28S rRNA | 0.372 | Tubulin | 0.749 | 0.41 | 1.42 |
|  | 6 | EF2 | 0.605 | EF2 | 0.442 | β-Actin | 0.637 | 0.64 | 2.01 |
|  | 7 | β-Actin | 0.667 | β-Actin | 0.494 | 28S rRNA | 0.5 | 0.26 | 1.05 |
| Different tissues of *P.americana* and *P.icosandra* | 1 | EF2 | 0.502 | EF2 | 0.04 | EF2 | 0.918 | 0.74 | 3.96 |
|  | 2 | EF1-α | 0.502 | EF1-α | 0.222 | EF1-α | 0.901 | 0.76 | 4.59 |
|  | 3 | 18S rRNA | 0.667 | 18S rRNA | 0.426 | 18S rRNA | 0.758 | 0.58 | 2.53 |
|  | 4 | β-Actin | 0.818 | β-Actin | 0.535 | 28S rRNA | 0.442 | 0.75 | 2.96 |
|  | 5 | Tubulin | 0.982 | 28S rRNA | 0.817 | β-Actin | 0.897 | 1.23 | 6.11 |
|  | 6 | 28S rRNA | 1.097 | Tubulin | 0.832 | GAPDH | 0.852 | 1.48 | 8.29 |
|  | 7 | GAPDH | 1.269 | GAPDH | 1.051 | Tubulin | 0.537 | 1.11 | 5 |
| Different tissues of *P.americana* and *P.acinosa* | 1 | 18S rRNA | 0.394 | 18S rRNA | 0.136 | EF2 | 0.748 | 0.66 | 3.47 |
|  | 2 | EF1-α | 0.394 | EF1-α | 0.211 | 18S rRNA | 0.69 | 0.46 | 2.03 |
|  | 3 | EF2 | 0.486 | β-Actin | 0.344 | EF1-α | 0.534 | 0.52 | 3.14 |
|  | 4 | β-Actin | 0.58 | EF2 | 0.412 | 28S rRNA | -0.599 | 0.56 | 2.25 |
|  | 5 | Tubulin | 0.678 | Tubulin | 0.46 | β-Actin | 0.898 | 6.12 | 23.9 |
|  | 6 | 28S rRNA | 0.734 | 28S rRNA | 0.536 | Tubulin | 0.888 | 3.7 | 14.6 |
|  | 7 | GAPDH | 0.99 | GAPDH | 1.07 | GAPDH | 0.154 | 1.28 | 7.37 |
| Different tissues of *P.icosandra* and *P.acinosa* | 1 | EF1-α | 0.439 | EF1-α | 0.133 | EF2 | 0.811 | 0.86 | 4.5 |
|  | 2 | GAPDH | 0.439 | GAPDH | 0.302 | EF1-α | 0.756 | 0.84 | 5.11 |
|  | 3 | EF2 | 0.649 | EF2 | 0.389 | 18S rRNA | 0.257 | 0.49 | 2.13 |
|  | 4 | 18S rRNA | 0.742 | 18S rRNA | 0.512 | GAPDH | 0.194 | 0.93 | 5.4 |
|  | 5 | β-Actin | 0.868 | β-Actin | 0.635 | 28S rRNA | 0.13 | 0.74 | 2.95 |
|  | 6 | Tubulin | 0.972 | Tubulin | 0.737 | β-Actin | 0.865 | 5.51 | 21 |
|  | 7 | 28S rRNA | 1.056 | 28S rRNA | 0.737 | Tubulin | 0.825 | 3.14 | 12.1 |
| Different tissues of three plant species | 1 | EF2 | 0.601 | EF1-α | 0.207 | EF2 | 0.803 | 0.78 | 4.09 |
|  | 2 | EF1-α | 0.601 | EF2 | 0.345 | EF1-α | 0.673 | 0.7 | 4.25 |
|  | 3 | 18S rRNA | 0.663 | 18S rRNA | 0.394 | 18S rRNA | 0.495 | 0.52 | 2.26 |
|  | 4 | β-Actin | 0.796 | β-Actin | 0.514 | 28S rRNA | -0.039 | 0.73 | 2.89 |
|  | 5 | Tubulin | 0.921 | Tubulin | 0.694 | β-Actin | 0.868 | 5.22 | 21.8 |
|  | 6 | 28S rRNA | 1.012 | 28S rRNA | 0.703 | Tubulin | 0.83 | 3.04 | 12.4 |
|  | 7 | GAPDH | 1.15 | GAPDH | 0.91 | GAPDH | 0.27 | 1.25 | 7.15 |

Table S2 continued

| Experimental conditions | rank | geNorm | | NormFinder | | BestKeeper | | | |
| --- | --- | --- | --- | --- | --- | --- | --- | --- | --- |
|  |  | gene name | stability value | gene name | stability value | gene name | r | SD | CV |
| Germinating seeds of three plant species | 1 | 28S rRNA | 0.373 | 18S rRNA | 0.06 | GAPDH | 0.791 | 0.61 | 3.59 |
|  | 2 | EF1-α | 0.373 | β-Actin | 0.067 | EF1-α | 0.6 | 0.27 | 1.59 |
|  | 3 | EF2 | 0.492 | EF2 | 0.142 | 18S rRNA | 0.438 | 0.16 | 0.71 |
|  | 4 | 18S rRNA | 0.577 | EF1-α | 0.15 | EF2 | 0.082 | 0.31 | 1.58 |
|  | 5 | β-Actin | 0.62 | 28S rRNA | 0.167 | 28S rRNA | 0.072 | 0.22 | 0.87 |
|  | 6 | Tubulin | 0.688 | Tubulin | 0.177 | Tubulin | 0.99 | 3.47 | 14 |
|  | 7 | GAPDH | 0.741 | GAPDH | 0.23 | β-Actin | 0.974 | 5.52 | 23.2 |
|  | 1 | EF1-α | 0.27 | EF1-α | 0.062 | EF1-α | 0.613 | 0.4 | 2.32 |
| Flowers of three plant species | 2 | GAPDH | 0.27 | 18S rRNA | 0.103 | 18S rRNA | 0.584 | 0.48 | 2.09 |
|  | 3 | EF2 | 0.295 | GAPDH | 0.119 | EF2 | 0.48 | 0.75 | 3.92 |
|  | 4 | Tubulin | 0.516 | 28S rRNA | 0.124 | β-Actin | 0.884 | 4.4 | 13.1 |
|  | 5 | 18S rRNA | 0.57 | Tubulin | 0.157 | Tubulin | 0.752 | 3.4 | 14 |
|  | 6 | 28S rRNA | 0.823 | EF2 | 0.168 | GAPDH | 0.407 | 1.02 | 5.68 |
|  | 7 | β-Actin | 1.003 | β-Actin | 0.222 | 28S rRNA | 0.2 | 1.2 | 4.66 |
|  | 1 | 18S rRNA | 0.314 | 18S rRNA | 0.062 | EF1-α | 0.939 | 0.56 | 3.35 |
| Different tissues of *S. litura*-infested *P.americana* | 2 | EF2 | 0.314 | EF2 | 0.079 | EF2 | 0.931 | 0.51 | 2.7 |
|  | 3 | EF1-α | 0.355 | EF1-α | 0.126 | β-Actin | 0.93 | 0.86 | 4.36 |
|  | 4 | β-Actin | 0.479 | β-Actin | 0.25 | 18S rRNA | 0.927 | 0.6 | 2.64 |
|  | 5 | Tubulin | 0.702 | Tubulin | 0.53 | Tubulin | 0.721 | 0.96 | 4.31 |
|  | 6 | 28S rRNA | 0.998 | 28S rRNA | 0.778 | 28S rRNA | -0.11 | 0.72 | 2.79 |
|  | 7 | GAPDH | 1.329 | GAPDH | 0.91 | GAPDH | 0.921 | 1.84 | 10.2 |
|  | 1 | 18S rRNA | 0.535 | EF1-α | 0.066 | EF1-α | 0.949 | 0.86 | 5.34 |
| Different tissues of *S. litura*-infested *P.icosandra* | 2 | β-Actin | 0.535 | EF2 | 0.094 | EF2 | 0.923 | 0.76 | 4.05 |
|  | 3 | EF1-α | 0.618 | 18S rRNA | 0.101 | 18S rRNA | 0.848 | 0.61 | 2.64 |
|  | 4 | EF2 | 0.685 | GAPDH | 0.124 | GAPDH | 0.817 | 0.84 | 4.72 |
|  | 5 | GAPDH | 0.731 | β-Actin | 0.142 | β-Actin | 0.775 | 0.78 | 3.93 |
|  | 6 | Tubulin | 0.822 | Tubulin | 0.22 | 28S rRNA | 0.503 | 0.89 | 3.59 |
|  | 7 | 28S rRNA | 0.971 | 28S rRNA | 0.285 | Tubulin | 0.877 | 1.25 | 5.42 |
|  | 1 | 18S rRNA | 0.462 | Tubulin | 0.179 | 28S rRNA | 0.561 | 0.34 | 1.39 |
| Different tissues of *S. litura*-infested *P.acinosa* | 2 | β-Actin | 0.462 | GAPDH | 0.298 | Tubulin | 0.551 | 0.61 | 2.1 |
|  | 3 | GAPDH | 0.506 | 28S rRNA | 0.477 | GAPDH | 0.536 | 0.68 | 4.06 |
|  | 4 | EF2 | 0.536 | β-Actin | 0.517 | β-Actin | 0.43 | 0.55 | 1.71 |
|  | 5 | 28S rRNA | 0.591 | 18S rRNA | 0.552 | 18S rRNA | 0.392 | 0.43 | 1.88 |
|  | 6 | Tubulin | 0.648 | EF2 | 0.746 | EF2 | 0.03 | 0.7 | 3.58 |
|  | 7 | EF1-α | 2.002 | EF1-α | 2.304 | EF1-α | 0.857 | 5.22 | 25.2 |
|  | 1 | 18S rRNA | 0.56 | 18S rRNA | 0.15 | EF1-α | 0.906 | 0.71 | 4.33 |
| Different tissues of *S. litura*-infested *P.americana* and *P.icosandra* | 2 | EF2 | 0.56 | EF1-α | 0.216 | EF2 | 0.891 | 0.64 | 3.4 |
|  | 3 | EF1-α | 0.597 | EF2 | 0.222 | β-Actin | 0.847 | 0.83 | 4.19 |
|  | 4 | β-Actin | 0.631 | β-Actin | 0.264 | 18S rRNA | 0.805 | 0.63 | 2.72 |
|  | 5 | Tubulin | 0.796 | Tubulin | 0.454 | 28S rRNA | 0.235 | 0.97 | 3.85 |
|  | 6 | 28S rRNA | 1.037 | 28S rRNA | 0.542 | GAPDH | 0.836 | 1.33 | 7.46 |
|  | 7 | GAPDH | 1.224 | GAPDH | 0.543 | Tubulin | 0.751 | 1.26 | 5.57 |
|  | 1 | 18S rRNA | 0.464 | 18S rRNA | 0.35 | 18S rRNA | 0.558 | 0.52 | 2.29 |
| Different tissues of *S. litura*-infested *P.americana* and *P.acinosa* | 2 | EF2 | 0.464 | β-Actin | 0.384 | EF2 | 0.491 | 0.62 | 3.22 |
|  | 3 | β-Actin | 0.535 | EF2 | 0.435 | 28S rRNA | -0.479 | 0.73 | 2.89 |
|  | 4 | Tubulin | 0.765 | Tubulin | 0.438 | Tubulin | 0.895 | 3.51 | 13.7 |
|  | 5 | 28S rRNA | 1.024 | GAPDH | 0.759 | β-Actin | 0.878 | 6.16 | 23.8 |
|  | 6 | GAPDH | 1.228 | 28S rRNA | 0.768 | EF1-α | 0.724 | 3.28 | 13.5 |
|  | 7 | EF1-α | 2.106 | EF1-α | 1.481 | GAPDH | 0.059 | 1.22 | 7.09 |

Table S2 continued

| Experimental conditions | rank | geNorm | | NormFinder | | BestKeeper | | | |
| --- | --- | --- | --- | --- | --- | --- | --- | --- | --- |
|  |  | gene name | stability value | gene name | stability value | gene name | r | SD | CV |
|  | 1 | β-Actin | 0.496 | GAPDH | 0.296 | EF2 | 0.587 | 0.79 | 4.15 |
| Different tissues of *S. litura*-infested *P.icosandra* and *P.acinosa* | 2 | 18S rRNA | 0.496 | 18S rRNA | 0.371 | 28S rRNA | 0.291 | 0.62 | 2.51 |
|  | 3 | EF2 | 0.595 | β-Actin | 0.412 | 18S rRNA | 0.181 | 0.54 | 2.31 |
|  | 4 | GAPDH | 0.656 | EF2 | 0.511 | GAPDH | -0.135 | 0.86 | 4.96 |
|  | 5 | Tubulin | 0.827 | 28S rRNA | 0.59 | Tubulin | 0.921 | 3.09 | 11.9 |
|  | 6 | 28S rRNA | 0.928 | Tubulin | 0.617 | β-Actin | 0.873 | 6.1 | 23.5 |
|  | 7 | EF1-α | 1.851 | EF1-α | 1.409 | EF1-α | 0.76 | 3.37 | 18.3 |
|  | 1 | 18S rRNA | 0.499 | 18S rRNA | 0.208 | EF2 | 0.606 | 0.7 | 3.7 |
| Different tissues of *S. litura*-infested three plant species | 2 | β-Actin | 0.499 | β-Actin | 0.25 | 18S rRNA | 0.399 | 0.56 | 2.44 |
|  | 3 | EF2 | 0.592 | EF2 | 0.39 | 28S rRNA | -0.05 | 0.82 | 3.25 |
|  | 4 | Tubulin | 0.851 | Tubulin | 0.615 | Tubulin | 0.906 | 2.96 | 11.9 |
|  | 5 | GAPDH | 1.048 | GAPDH | 0.869 | β-Actin | 0.874 | 5.45 | 22.8 |
|  | 6 | 28S rRNA | 1.202 | 28S rRNA | 0.948 | EF1-α | 0.753 | 2.41 | 13.5 |
|  | 7 | EF1-α | 1.885 | EF1-α | 2.415 | GAPDH | 0.082 | 1.17 | 6.72 |





**Figure S1. Length distribution of protein sequences transcript from the coding sequences predicted.**

**

**

**Figure S2. Classification of the transcripts annotated by Clusters of Orthologous Groups of proteins (COG).**

**

**

**Figure S3. Classification of the transcripts annotated by evolutionary genealogy of genes: Non-supervised Orthologous Groups (eggNOG).**

**

**

**Figure S4. Classification of the transcripts annotated by euKaryotic Ortholog Groups (KOG).**
